# Supplementary material for: Effect of balance training on footwork performance in badminton: An interventional study
Source: PLoS One. 2022 Nov 17;17(11):e0277775. doi: 10.1371/journal.pone.0277775 (PMC9671355; doi:10.1371/journal.pone.0277775)
Supplement: S1 File — (PDF) [file pone.0277775.s001.pdf]

S1 File - Balance training protocol

Week	Balance Intervention	Description	Time allocation
**1-2**	Tandem walking along the badminton court.	**Exercise 1**<br>Participant was holding the badminton racket in the dominant hand keeping the forearm pronated, elbows extended, shoulder abducted to 90 degrees. Participant was instructed to do tandem walking along the boundary line of the badminton court for 3 minutes. Then the exercise was repeated with shoulder flexed to 90 degrees for another 2 minutes.<br><br>**Exercise 2**<br>Participant was holding the badminton racket in the dominant hand with shuttlecock placed on the racket. Participant was instructed to keep the forearm supinated, elbows extended, shoulder abducted to 90 degrees. Then the participant was instructed to do tandem walking along the boundary line of the badminton court for 3 minutes. The exercise was progressed by instructing the participant to follow the	5 minutes<br><br>5 minutes

		same exercise with shoulder flexed to 90 degrees for another 2 minutes.	
	Alternating rotational lunges	Participant was instructed to stand with both legs hip-width apart and keep both arms straight out in front of him. Participant was asked to step one foot forward into a lunging position, bend both knees and rotate arms and trunk over the lead leg, rotate back to neutral and stand up. Then the participant was instructed to do the same procedure with the opposite leg.	8 minutes (10 repetitions for each direction of the exercise with 30 seconds of rest at the end of 10 repetitions)
	Double-legs standing on the both-sides up (BOSU) balance trainer	**Exercise 1**<br>Participant was instructed to stand with both legs with shoulder-width apart on the inflated rubber hemispheric surface of the BOSU and maintain balance with minimum fluctuations of center of gravity (COG) over the base of support (BOS). Participant was instructed to perform the same exercise with a smaller BOS by moving the legs closer.<br><br>**Exercise 2**<br>Participant was instructed to stand with both legs with shoulder-width apart on the rigid flat surface of the BOSU and maintain balance with minimum	3 minutes<br><br>3 minutes

		fluctuations of COG over the BOS. Participant was instructed to perform the same exercise with a smaller BOS by moving the legs closer.	
	Double-legs standing on the Wobble board	Participant was instructed to stand with both legs with shoulder-width apart on the flat surface of the Wobble board and maintain balance with minimum fluctuations of COG over the BOS.	6 minutes
**3-4**	Horizontal lunge to balance	Participant was instructed to stand straight with both feet hip-width apart and keep both arms on the waist. Participant was asked to step one foot sideways into a lunge position and come back to the starting position. Then the participant was instructed to repeat the exercise with the opposite leg.	8 minutes<br>(10 repetitions for each direction of the exercise with 30 seconds of rest at the end of 10 repetitions)
	Single-leg dead lift and reach	Participant was instructed to stand straight with both feet and arms holding straight to 90 degrees. Participant was asked to shift the weight to the supporting leg, as he slide his non-supporting leg back, allowing his upper body to move forward as the hinge. Participant was instructed to keep bending forward until it was almost parallel to the floor while keeping his arms straight. Participant was expected to keep the support leg and the non-supporting leg straight while	8 minutes<br>(10 repetitions for each direction of the exercise with 30 seconds of rest at the end of 10 repetitions)

		doing the exercise. Then the participant was instructed to do the same procedure with the opposite leg.	
	Single-leg standing on the BOSU	**Exercise 1**<br>Participant was instructed to stand with one legs on the inflated rubber hemispheric surface of the BOSU and maintain balance with minimum fluctuations of COG over the BOS and repeat the exercise with the opposite leg.<br><br>**Exercise 2**<br>Participant was instructed to stand with one leg on the rigid flat surface of the BOSU and maintain balance with minimum fluctuations of COG over the BOS and repeat the exercise with the opposite leg.	3 minutes<br><br>3 minutes
	Double-legs standing on the Wobble board	Participant was instructed to stand with both legs at shoulder-width apart on the flat surface of the Wobble board with and maintain balance with minimum fluctuations of COG over the BOS. Participant was instructed to perform the same exercise with a smaller BOS by moving legs closer.	6 minutes
**5-6**	Single-leg skater jumps	Participant was standing with both legs hip-width apart. Then the participant was instructed to bend one leg behind the supporting leg at a slight angle, while	10 minutes (20 repetitions with 30 seconds of

		maintaining weight and balance on the supporting leg. Then the participant was asked to simultaneously swing his arms out and jump laterally and land over on the opposite side shifting his weight and allowing the non-supported leg to cross behind the supported leg. The participants was expected to land on the forefoot with his hips and knees bent slightly. Participant was instructed to repeat this side-to-side motion, transferring the weight each time.	rest at the completion of 10 repetitions)
	Lunging in longitudinal, diagonal and transverse directions on BOSU	**Exercise 1**<br>Participant was standing on the floor with both legs hip-width apart, keeping both arms on the waist. Participant was instructed to step one foot in the longitudinal direction into a lunge position on the inflated rubber hemispheric surface of BOSU and come back to the starting position. Then the participant was instructed to do the same procedure with the opposite leg.<br><br>**Exercise 2**<br>Participant was standing on the floor with both legs hip-width apart, keeping both arms on the waist. Participant was asked to step one foot in the diagonal	5 minutes (10 repetitions with 30 seconds of rest at the end perform the exercise with the opposite leg with same number of repetitions and rest period)<br><br>5 minutes (10 repetitions with 30 seconds of rest at the end

		direction into a lunge position on the inflated rubber hemispheric surface of BOSU and come back to the starting position. Then the participant was instructed to do the same procedure with the opposite leg.<br><br>**Exercise 3**<br>Participant was standing on the floor with both legs hip-width apart, keeping both arms on the waist. Participant was asked to step one foot in the transverse direction into a lunge position on the inflated rubber hemispheric surface of BOSU and come back to the starting position. Then the participant was instructed to do the same procedure with the opposite leg.	perform the exercise with the opposite leg with same number of repetitions and rest period)<br><br>5 minutes<br>(10 repetitions with 30 seconds of rest at the end perform the exercise with the opposite leg with same number of repetitions and rest period)
	Single-leg standing on Wobble board	Participant was instructed to stand with one leg on the flat surface of the Wobble board and maintain balance with minimum fluctuations of COG over the BOS. Participant was instructed to perform the same exercise with the opposite leg.	5 minutes

**7-8**	Single-leg rotational skater jumps	Participant was standing with both legs hip-width apart. Participant was instructed to bend the leg behind the supporting leg at a slight angle, while maintaining weight and balance on the supporting leg. Then the participant was asked to simultaneously swing his arms out and jump laterally, land over on the opposite side. As the participant jump to the opposite side, he was asked to rotate the upper body shifting his weight and allowing the non-supported leg to cross behind the supported leg. The participants was instructed to land on the forefoot with his hips and knees bent slightly. Participant was instructed to repeat this side-to-side motion, transferring the weight each time.	10 minutes (20 repetitions with 30 seconds of rest at the completion of 10 repetitions)
	Longitudinal, diagonal and transverse step catch on BOSU	**Exercise 1** Participant was standing on the floor with both legs hip-width apart. Participant was instructed to jump and land on the inflated rubber hemispheric surface of BOSU in one foot in the longitudinal direction. Then the participant was instructed to do the same procedure with the opposite leg. **Exercise 2** Participant was standing on the floor with both legs hip-width apart. Participant was instructed to jump and	4 minutes (10 repetitions for each exercise with 30 seconds of rest at the end of 10 repetitions) 4 minutes (10 repetitions for each exercise with

		land on the inflated rubber hemispheric surface of BOSU in one foot in the diagonal direction. Then the participant was instructed to do the same procedure with the opposite leg.<br><br>**Exercise 3**<br>Participant was standing on the floor with both legs hip-width apart. Participant was instructed to jump and land on the inflated rubber hemispheric surface of BOSU in one foot in the transverse direction. Then the participant was instructed to do the same procedure with the opposite leg.	30 seconds of rest at the end of 10 repetitions)<br><br>4 minutes<br>(10 repetitions for each exercise with 30 seconds of rest at the end of 10 repetitions)
	Hitting the shuttlecock while standing on BOSU	**Exercise 1**<br>Participant was standing on both legs, shoulder width-apart on the rubber hemispheric surface of the BOSU. Then the participant was instructed to successfully hit a series of shuttlecocks which were fed continuously by a co-player.<br><br>**Exercise 2**<br>Participant was standing on both legs shoulder width-apart on the rigid flat surface of the BOSU. Then the participant was instructed to successfully hit a series of	2 minutes<br><br>2 minutes

		shuttlecocks which were fed continuously by a co-player.	
	Single-leg standing on Wobble board	Participant was instructed to stand with one leg on the flat surface of the Wobble board and maintain balance with minimum fluctuations of COG over the BOS. Participant was instructed to repeat the exercise with the opposite leg.	4 minutes
