# Supplementary material for: Effect of balance training on footwork performance in badminton: An interventional study
Source: PLoS One. 2022 Nov 17;17(11):e0277775. doi: 10.1371/journal.pone.0277775 (PMC9671355; doi:10.1371/journal.pone.0277775)
Supplement: Attachment — Submitted filename: Response to Reviewers.docx [file pone.0277775.s002.docx]

04.10.2022

Prof. Emily Chenette,

The Editor-in-Chief

‘PLoS ONE’

Dear Prof. Chenette,

**Submission of the revised manuscript ‘Effect of balance training on footwork performance in badminton: an interventional study’**

As the corresponding author, I thank the academic editor and the two reviewers for their comments on our manuscript. Below is our response to each point raised by the academic editor and reviewers. We hope that we satisfyingly addressed them and that the manuscript will be now suited for publication.

Thank you,

Your Sincerely,

Kavinda Malwanage,

Corresponding author,

Faculty of Allied Health Sciences,

University of Peradeniya,

Peradeniya,

Sri Lanka

Reviewer #1: The approach of the paper is interesting because it examines the effect of balance training on footwork performance in badminton but I think the authors should respond to the comments below in order to improve the quality of the paper. Despite I think the authors should increase the number of the sample (they have use only 8 in control group and 12 in the intervention group) I propose a major revision of the manuscript to be considered for publication (see the following comments).

In the introduction section important references are missed. In the first paragraph when discussing the timing structure in badminton are missed 3 important references (i.e. Abian-Vicen et al., 2013; Abián, 2014; Abián-Vicén et al., 2018). In the second paragraph when are talking about that lunges place high physical demands on the lower limbs are missed 2 important references (ie. Bravo-Sánchez et al, 2019; Bravo Sánchez et al., 2019). These articles should be included.

Abian-Vicen J, Castanedo A, Abian P, & Sampedro J. (2013). Temporal and notational comparison of badminton matches between men's singles and women's singles. Int J of Perf Anal Sport, 13(2), 310-320.

Abián P, Castanedo A, Feng XQ, Sampedro J, & Abian-Vicen J. (2014). Notational comparison of men's singles badminton matches between Olympic Games in Beijing and London. Int J of Perf Anal Sport, 14(1), 42-53

Abián-Vicén J, Sánchez L, & Abián P. (2018). Performance structure analysis of the men’s and women’s badminton doubles matches in the Olympic Games from 2008 to 2016 during playoffs stage. International Journal of Performance Analysis in Sport, 1-12. doi: 10.1080/24748668.2018.1502975

Bravo-Sánchez, A., Abián, P, Jiménez, F, Abián-Vicén, J. (2019). Myotendinous asymmetries derived from the prolonged practice of badminton in profesional players. Plos One, 14(9): e0222190. doi: 10.1371/journal.pone.0222190

Bravo-Sánchez, A, Abián-Vicén, J, Jiménez, F, Abián, P. (2019). Influence of badminton practice on calcaneal bone stiffness and plantar pressure. Physician and Sportmedicine, 48(1), 98-104. doi: 10.1080/00913847.2019.

Response:

We acknowledge that the findings of the above-mentioned studies add important evidence to set the background for our study. Therefore, the suggested references have been added to the relevant sections of the revised manuscript in Introduction > pages 4 and 5

The badminton training should be explained to both groups to know the difference exercises in the control group during the 30 minutes that is longer the session of badminton training compared to the intervention group.

Response:

In this study, the intervention group underwent 30-minute balance training and 1 hour and 30 minutes of ordinary badminton training whereas the control group underwent 2-hour ordinary badminton training each day, under one coach. After the 30 minutes of balance training, the intervention group started off the ordinary badminton training as they are starting a badminton session under the same coach on a regular training day. As such, the 30 minutes they lost was from the latter end of the practice session, where the control group players used to play practice matches, etc. with their playmates. Therefore, the intervention group also had similar skills training as the control group. This has been now explained in the revised manuscript in Materials and methods > Study protocol and intervention > lines 176-179.

The shuttle run test and the testing for footwork performance have not been validated for any author.

Response:

The ‘Shuttle run test’ which was mentioned in lines 131 and 230 in the former version of the manuscript seems to give a wrong impression that it is a test that needs validation against an on-court performance (e.g. generic tests of balance). Rather, the shuttle run is a coaching phenomenon and practice-based protocol which does not have roots in science but in badminton practice (Badminton World Federation coaching manuals). Specifically, the shuttle run mimics sport-specific footwork and therefore best considered a proxy measure of badminton footwork performance. The shuttle run routine used in this study involved picking and placing the shuttles from/on pre-defined corners/sides of the court and thus was uniform across sessions and participants. This has been clarified in a shuttle run description in Materials and methods > Shuttle run > line 250-252.

It is impossible to know that the shuttle is hit for all the participant in the same point (zone) during the testing for footwork performance. The shuttle is feeded by a person and in variables recorded in milliseconds the shuttle should be exactly in the same point for all the participants.

Response:

Although the shuttles were fed randomly, it was made sure that each shuttle was fed to each corner of the six primary locations (zones) of the court. Therefore, as far as the corner of the court is considered, each participant had to go to each corner/zone the same number of times during a session. In individual shots, the exact point in court the shuttle was supposed to land could not be strictly controlled because of the human error factor in the coach who fed the shuttles. Therefore, although the shuttle was fed to that particular corner, the exact point where the shuttle was contacted varied among different strokes, and this in turn may have affected the push-off times (e.g. Shuttles that are fed to the extreme corner, players tend to take longer steps with longer durations to get recovered to maintain balance). However, we believe that this variation was random and it was not systematically biased towards one participant, session or a group. This information is noted in Materials and methods > Measurement of push-off time in stroke play > lines 263-270 of the revised manuscript. This random variability manifests as a reduced precision and increased standard deviations (SD) of push-off times in individuals. This increased variability, and in turn SD around the mean push-off time has been acknowledged as a limitation in the Discussion > lines 385-387.

In the results of the tests (in the tables) you can see that not all the participants have carried out all the tests.

Response:

All participants could complete all balance tests. However static balance with eyes closed data of one participant from the intervention group and one participant from the control group had to be excluded because of being outliers (Table 2).

Shuttle run data are based on all participants.

Footwork performance data were incomplete for some shots and sessions because the participants either (1) failed to perform desired badminton shot (e.g., perform another shot instead of the rear forehand shot), or (2) unable to perform an effective shot (i.e., hit the shuttlecock with badminton racket). Consequently, the numbers completed each stroke (in Table 3) are different. Particularly this caused a major reduction in samples for front backhand, side backhand and rear forehand shots. Therefore, causing less number of data under these badminton shots. These data are also presented with the rest, but we did not make any interpretations based on those limited data. This information has now been included in the revised manuscript in Materials and methods > Statistical analysis > lines 282-290.

In table 3 in some variables there are 1 or 2 participants???. That is wrong.

Response:

The reason for this is explained in the above response. This marked reduction in samples is acknowledged as a limitation in the revised manuscript in Discussion > lines 385-387.

The discussion of the results should be done with more rigor and consistency. Also should be longer.

Response:

We have only discussed the most important results. We focused to discuss the results (i.e., significant improvement of static and dynamic balance, shuttle run time and push-off times between groups) based on the p value of the time*group interaction effect. Significant p values either for time or group effect were not considered as we believed that time*group interaction overwhelm the sole effect of either the group effect (i.e., intervention vs control) or the time effect (i.e. pre vs post) to improve the balance and footwork performance in badminton players. However, in the discussion of the revised manuscript, we have added a description on non-significant outcomes and included a paragraph discussing the limitations of our study in Discussion > lines 351-352, 380-389.

For all the above comments I propose a major revision.

Reviewer #2: Comments to the authors

The proposed manuscript is very interesting and has a noteworthy objective. Congratulations to the authors. My suggestions follow below.

Abstract: In the phrase “Balance training is an unexplored component….”, do the authors mean that “Balance training has not been sufficiently explored…?” There are already some studies in this field.

Response:

We meant to say that the inclusion of balance training in badminton is not widely adopted even though the efficacy of balance training in injury prevention and performance enhancement is already explored up to some extent in the field of sport. We have revised the manuscript according to this comment and the first comment of Reviewer #1.

Introduction > pages 4,5,6

Keywords: I suggest not repeating the keyword that already exists in the title to enhance search results.

Response:

This has been changed in the revised manuscript: lines 46-47. Accordingly, the modified keywords are badminton, balance training, interventional study, push-off time, shuttle run, sports performance.

The two phrases contained in lines 79-82 require references. The same goes for other sentences without references, specifically in the introduction section.

Response:

The references have been added in line 81 in the revised manuscript and other sentences in Introduction > pages 4,5,6.

The paragraph starting at line 79 and ending at 102, is very interesting for the study to point out the importance of balance in badminton, however the transition between the studies should be further explored so that it's clearer for the reader.

Response:

A bridging section has been inserted into the Introduction (lines 121-123, 130-133), to allow a smooth transition.

Line 103: “footwork performance” is core in your study, however, no reference in the introduction exists to introduce the importance of “footwork performance” neither how it is assessed. I consider its essential to add a paragraph to support “footwork performance” importance and assessment.

Response:

In the introduction section, we have mainly described the characteristics of badminton, the importance of balance in badminton, and footwork performance in badminton. The section on the importance, kinetics and kinematics of badminton-specific footwork has been included in the Introduction along with the pertinent references. Lines 90-101.

Line 114: This is not an RCT. It may however be a clinical trial with a convenience sample which was randomized in to two groups.

Response:

The nature of the study populations (i.e. sportsmen in a squad) precluded us from randomly recruiting the subjects, but group assignment (control vs, intervention) was randomized. However, to minimize confusion, revised the manuscript by replacing ‘randomized controlled trial’ with ‘controlled trial’: Abstract > line 28, Materials and methods > Study design and setting > line 141, Conclusions > line 391. In this study, we have conducted a balance training intervention (which is not a health care intervention), and the outcomes we measured (static balance, dynamic balance, shuttle run time, push-off time) are performance-related outcomes (not health-related outcomes). Therefore, we did not register the trial in the clinical trials registry as it could not be considered a clinical trial.

Was a sample size calculated? Please state this in the manuscript.

Response:

The selection of the study sample was largely governed by the availability of resources and the authors’ ability to conduct the balance training program to a number of participants in a closely supervised manner. We also believed that it is impossible to train a large number of players at a time by one coach in a closely supervised manner. We wanted to control for interprofessional variability which could occur as a result of different coaching styles. Therefore, we did not include different badminton teams who were practicing under different coaches as the performance of each group can be varied according to the coaching style. The largest cohort of players that we could find was a pool of 30 adolescent badminton players, in which 15 players were included in the intervention group and 15 players were included in the control group. This was the possible sample size that we could access without compromising interrater variability/ inter coach variability. Still, we believe based on our results this training and assessment model can be implemented in larger, elite-level cohorts of badminton. In that sense, the present trial would also serve as a proof-of-concept study. This has been mentioned in the revised manuscript in Discussion > lines 380-385.

Was data protection guaranteed? Please state this detail on the manuscript.

Response:

The authors guaranteed to preserve the confidentiality of the participants and protect the collected data. This information has been stated in the revised manuscript in Materials and methods > Participants > lines 159-161.

Line 176: Please state the reference used “Each participant’s dominant leg was identified as the leg which moved close to the racket arm when lunging”.

Response:

In badminton, the dominant leg is referred to as the leg that moves close to the racket arm when lunging. The racket arm could be identified as the arm used by the player to hold the racket to reach the shuttle and hit. Therefore, in our study also we identified the dominant leg as the leg moved close to the racket arm when lunging. There is no historical scientific reference for the dominant leg in badminton but it comes from the regular practice in badminton and coaching manuals. The definition has been used extensively in BWF coaching manuals (<http://www.badminton-israel.co.il/newsNdata/General/CoachEducationBWF/BWF_Coach_Manual_Level_1.pdf>, <https://czechbadminton.cz/files/autor/bwf_coach_education_coaches_manual_l2_lowres.pdf>) and by previous researchers (Abián-Vicén et al., etc.)

Line 216: Please state the reference for the “Shuttle Run Test” used in the study.

Response:

This response is related to a very similar response made above to a comment by Reviewer #1. The ‘Shuttle run test’ which was mentioned in lines 131 and 230 in the former version of the manuscript seems to give a wrong impression that it is a test that needs validation against an on-court performance (e.g. generic tests of balance). Rather, the shuttle run is a coaching phenomenon and practice-based protocol which does not have roots in science but in badminton coaching and practice, also compiled in current BWF coaching manuals for instance (<http://www.badminton-israel.co.il/newsNdata/General/CoachEducationBWF/BWF_Coach_Manual_Level_1.pdf>, <https://czechbadminton.cz/files/autor/bwf_coach_education_coaches_manual_l2_lowres.pdf>) Specifically, the shuttle run mimics sport-specific footwork and therefore best considered a proxy measure of badminton footwork performance. The shuttle run routine used in this study involved picking and placing the shuttles from/on pre-defined corners/sides of the court and thus was uniform across sessions and participants. This has been clarified in a shuttle run description in Materials and methods > Shuttle run > lines 250-252.

Line 225: Please state the reference for the “Measurement of push-off time in stroke play”.

Response:

In badminton, the player is required to respond to his opponent’s stroke with appropriate footwork, and appropriate positioning of the body, execute a return stroke then recover to the most appropriate court position during the match. Therefore, basic split-step, lateral split-step, and diagonal split-step are trained by the badminton players to improve their push-off footwork performance and balance Further, the players are required to perform an explosive push-off from the ground on landing and make the ground contact time short to move off quickly in response to the opponent’s shot (<http://www.badminton-israel.co.il/newsNdata/General/CoachEducationBWF/BWF_Coach_Manual_Level_1.pdf>, <https://czechbadminton.cz/files/autor/bwf_coach_education_coaches_manual_l2_lowres.pdf>). Therefore, in our study, we measured this performance-related skill based on the push-off time as it is encouraged to extensively practice during training and real gameplay. This aspect appears repeatedly in different sections of the manuscript. Measurement of push-off time through high-frame-rate video recording in not only specific to badminton push-off times but to many speeded maneuvers in sports.

Statistical analysis

Please state if parametric or non-parametric test were used in each data set analysis.

Response:

We have mentioned the name of the statistical test used in the analysis (Two-way, group (intervention vs. control) × time (pre vs. post) mixed analysis of variance (ANOVA)) in the manuscript in Methods and materials > Statistical analysis > lines 291-293. This is a parametric test.

Line 257: Please add P value calculation and if possible confidence intervals to “Table 1. Sample characteristics.”

Response:

The p values and confidence intervals have been included in Table 1 in the revised manuscript in

Results > Sample characteristics > Table 1> line 311.

Line 266: It is not correct to refer a “trend” in the phrase “There was a trend for a time main effect (p = 267 0.114) and time x group interaction (p = 0.085) in shuttle run time:” It is simply not significant.

Response:

The phrase has been removed from the revised manuscript as the literature also suggests not using the term ‘trend’ to describe non-significant effects. Results > Balance performance and shuttle-run time.

Please state the limitations of the study in the manuscript.

Response:

We have added the limitations of the study in the revised manuscript under Discussion > lines 380-389.

Conclusion may need reformulation as the sample size is considerably small and fact that this study is not a RCT.

Response:

This has been mentioned in Conclusion > line 391 and acknowledged in the penultimate paragraph of the Discussion > line 380-385.

Flow diagram seems incorrect in the Allocation section.

Response:

The flow diagram of the Fig 1 is edited accordingly in the revised manuscript.

Was the trial registered in a clinical trials platform?

Response:

According to the clinical trials registry in the country in which this research was conducted, a clinical trial is defined as ‘any research that prospectively assigns human subjects (patients or healthy volunteers) to one or more healthcare interventions’. A 'health care intervention' can be a drug, vaccine, surgical treatment, device, behavioral modification, preventive measure or a process of care change, that would lead to a change in a health-related outcome’. Any trial meeting the above standards are required to get registered, and the registration requires the clinical condition under question and the phase of trial. Accordingly, the clinical condition of focus should be specified in the registry application before proceeding to the completion of submission. The balance training intervention we conducted was oriented to improve performance (rather than a health-related outcome), and the outcomes we measured (static balance, dynamic balance, shuttle run time, push-off time) are sport-performance-related outcomes (not health-related outcomes). Therefore, we did not register the trial in the clinical trials registry. This is similar to many other sports-performance outcome trials conducted elsewhere.
